# Supplementary material for: Pyroptosis inhibiting nanobodies block Gasdermin D pore formation
Source: Nat Commun. 2023 Dec 1;14:7923. doi: 10.1038/s41467-023-43707-z (PMC10692205; doi:10.1038/s41467-023-43707-z)
Supplement: Supplementary file 1 — Supplementary Information [file 41467_2023_43707_MOESM1_ESM.pdf]

Supplementary Information

Pyroptosis inhibiting nanobodies block Gasdermin D pore formation

Anja Kopp[1,2], Gregor Hagelueken[1], Isabell Jamitzky[1], Jonas Moecking[1],
Lisa D.J. Schiffelers[3], Florian I. Schmidt[3] & Matthias Geyer[1,*]

[1] Institute of Structural Biology, University of Bonn, Venusberg-Campus 1, 53127 Bonn, Germany.

[2] Inflammation Division, The Walter and Eliza Hall Institute of Medical Research, 12 Parkville, VIC 3052, Australia.

[3] Institute of Innate Immunity, University of Bonn, Venusberg-Campus 1, 53127 Bonn, Germany.

* Correspondence should be addressed to: matthias.geyer@uni-bonn.de

The PDF file includes:

Supplementary Figures S1 – S6

Supplementary Tables 1 and 2

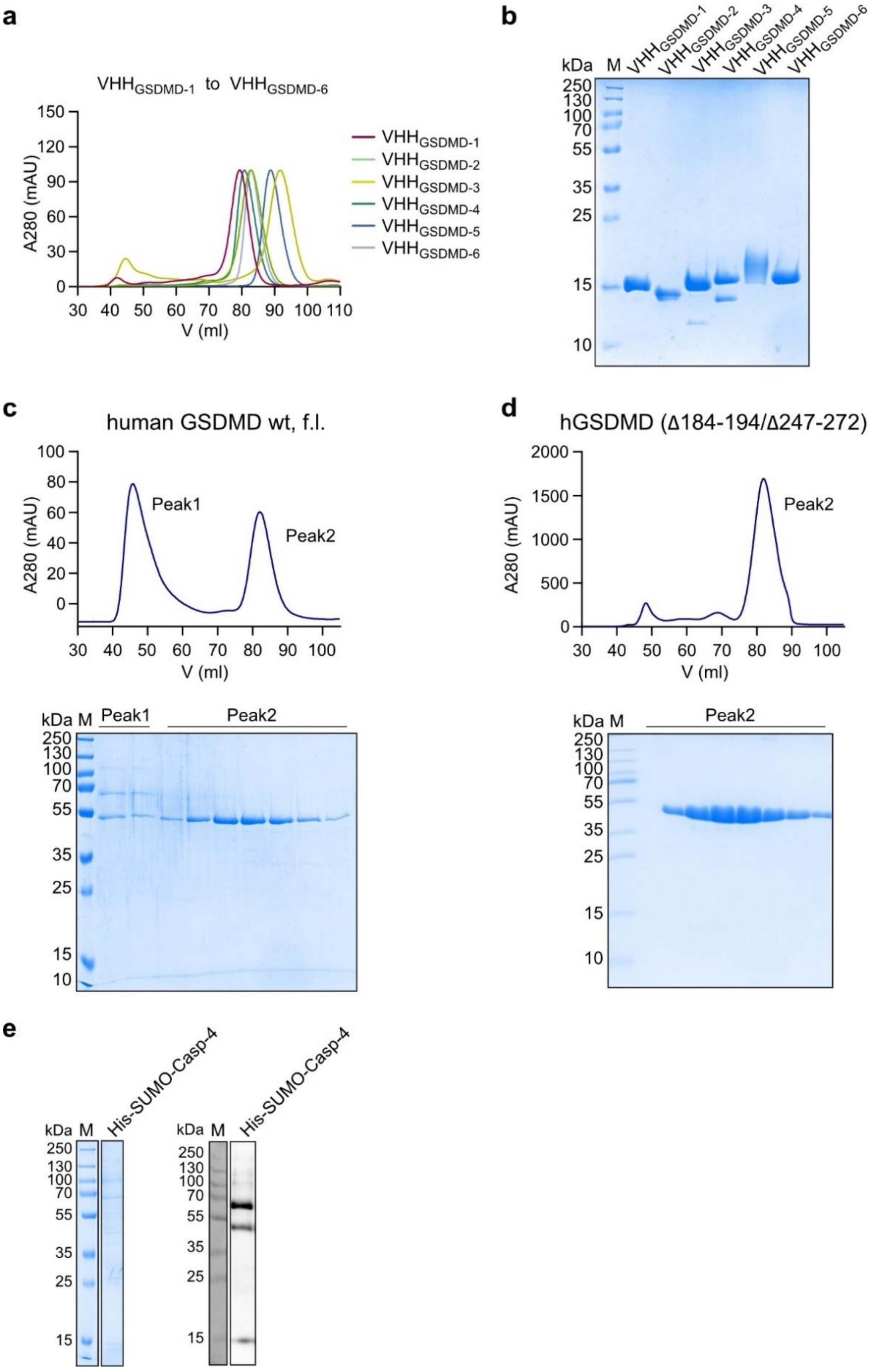

Supplementary Fig. 1 | Nanobodies and GSDMD variants used in this study. a, SEC elution chromatogram and **b**, SDS-PAGE of the recombinantly expressed and purified nanobodies $VHH_{GSDMD-1}$ to $VHH_{GSDMD-6}$. **c**, SEC elution chromatogram and SDS-PAGE analysis of wild type, full length, human GSDMD (1-484). **d**, SEC elution chromatogram and SDS-PAGE analysis of a human GSDMD variant (1-484; residues 184-194 and 247-272 were deleted). **e,** SDS-PAGE of recombinant His-SUMO-Caspase-4 and western blot of His-SUMO-Caspase-4 using an anti-His-tag antibody. Source data are provided as a Source Data file.

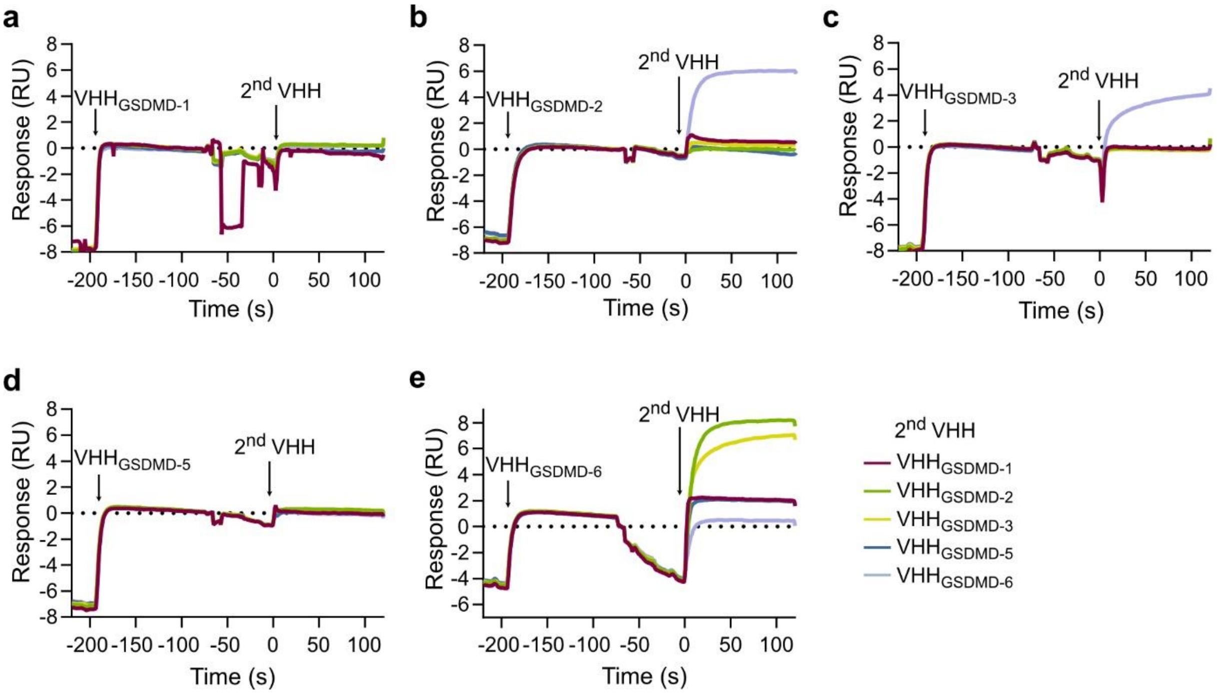

Supplementary Fig. 2 | SPR-based epitope binning of VHH$_{GSDMD}$ binding. a, Epitope binning assay with VHH$_{GSDMD-1}$ submitted in a first titration step followed by a second titration step with one nanobody of the pool of five (VHH$_{GSDMD-1}$, $_{-2}$, $_{-3}$, $_{-5}$, and $_{-6}$). Chemically biotinylated human, full length GSDMD was immobilized on an SPR sensor chip and the competitive binding of nanobodies was tested in a pair-wise manner. **b**, Epitope binning assay with VHH$_{GSDMD-2}$ submitted in the first titration step. Association of the second nanobody to a distinct epitope can be observed for VHH$_{GSDMD-6}$ as a second association event in the SPR sensorgram. **c-e**, Same as in **a** starting with VHH$_{GSDMD-3}$, $_{-5}$, and $_{-6}$, respectively. Source data are provided as a Source Data file.

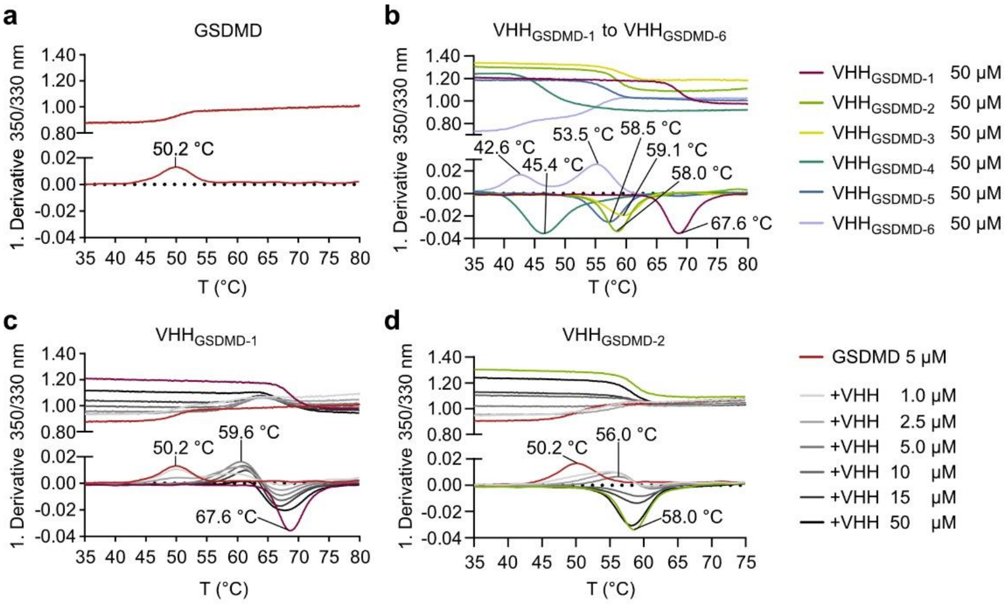

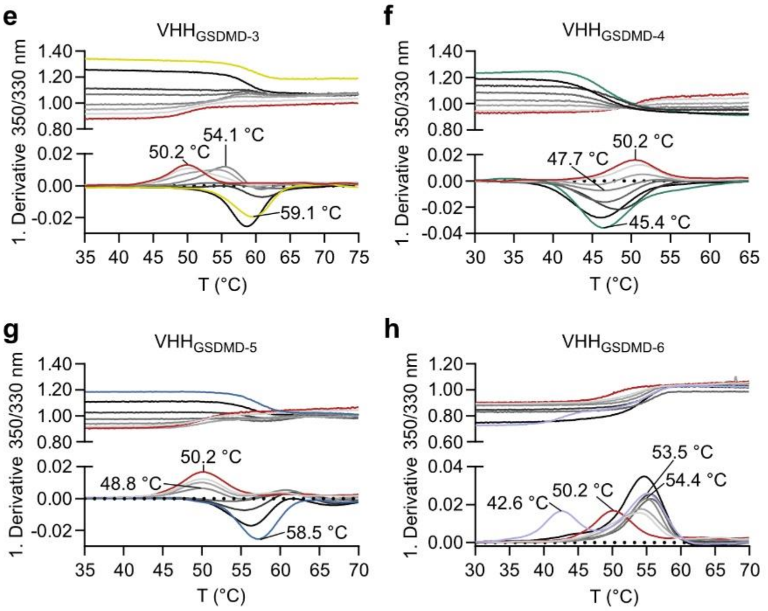

i

Sample	Tm (°C)	SD
GSDMD 5 µM	50.2	0.3
VHH-1 50 µM	67.6	1.2
VHH-2 50 µM	58.0	0.5
VHH-3 50 µM	59.1	1.8
VHH-4 50 µM	45.4	2.2
VHH-5 50 µM	58.5	2.9
VHH-6 50 µM Tm_1	42.6	0.2
VHH-6 50 µM Tm_2	53.5	1.3
+VHH-1 5 µM	59.6	1.0
+VHH-2 5 µM	56.0	1.0
+VHH-3 5 µM	54.1	1.3
+VHH-4 5 µM	47.7	2.5
+VHH-5 5 µM	48.8	0.8
+VHH-6 5 µM	54.4	0.5

Supplementary Fig. 3 | Effect of the nanobodies on the thermal stability of GSDMD. a, Melting temperature of human GSDMD at a concentration of 5 µM. The melting temperature (T_m) was determined using nano-differential scanning fluorimetry (nanoDSF). **b**, Melting temperatures of the six nanobodies alone at a concentration of 50 µM. **c-h**, Melting temperatures of the GSDMD–nanobody complexes at varying nanobody concentrations. For the titration experiment, 5 µM GSDMD was mixed with increasing concentration of the respective nanobody in a range of 1-50 µM. **i**, Summary of melting temperatures of GSDMD only, VHHs, and GSDMD–VHHs complexes at indicated concentrations. Source data are provided as a Source Data file.

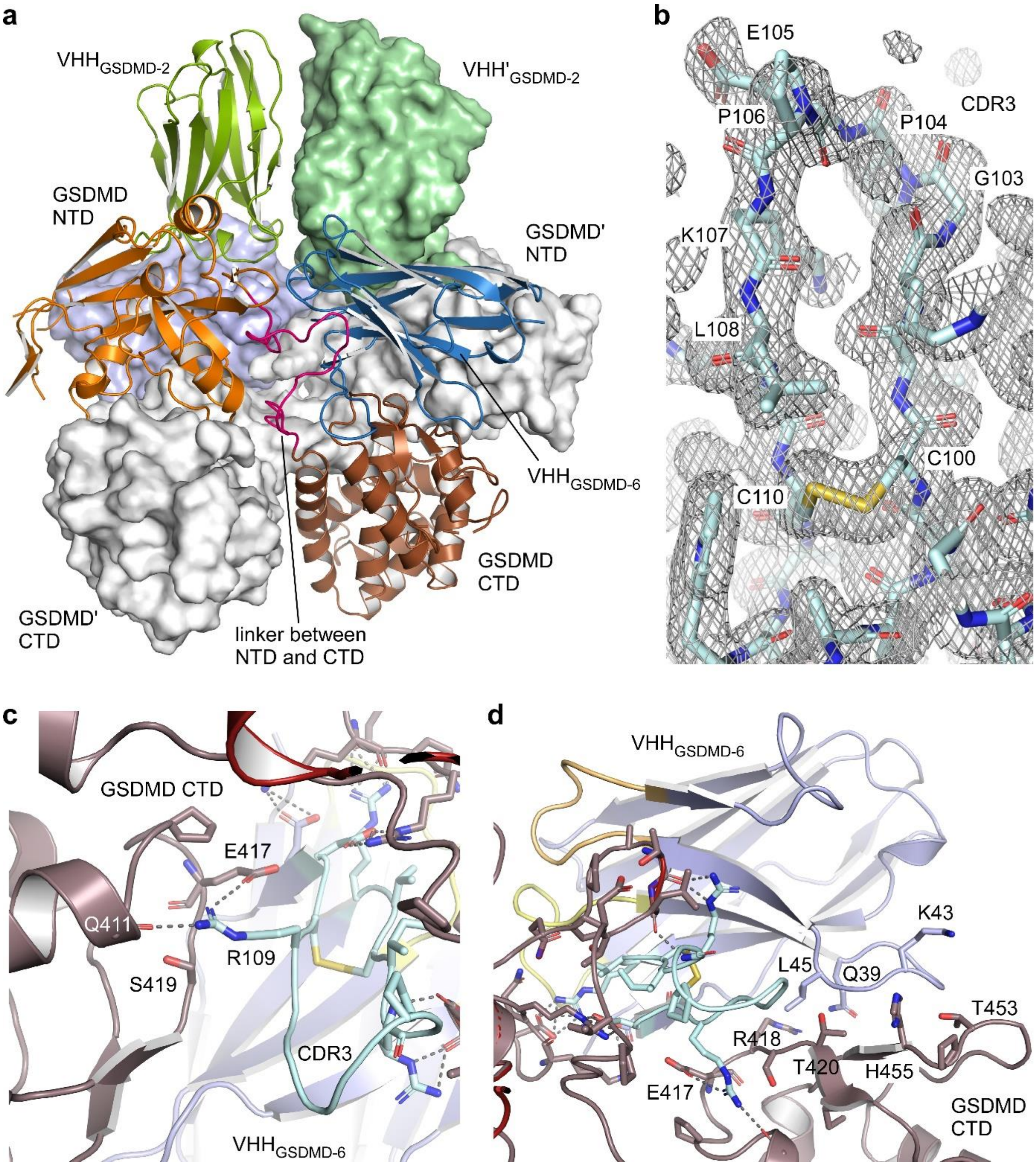

Supplementary Fig. 4 | Details for the GSDMD–nanobody interactions. a, The N- and C-terminal domains of GSDMD are twisted in the dimer assembly of the ternary GSDMD–$VHH_{GSDMD-2}$–$VHH_{GSDMD-6}$ complexes. One trimeric complex (chains A, B, C) is shown in ribbon representation while the other one is shown as surface representation. **b**, Electron density map ($2mF_o$-DF_c) of the C100–C110 disulfide bond and the entire CDR3 of $VHH_{GSDMD-6}$ at 1.0 σ. **c**, Salt bridge interactions of $VHH_{GSDMD-6}$ CRD3 to the GSDMD CTD. **d**, Hydrophobic interactions stabilize the binding of $VHH_{GSDMD-6}$ to the CTD of GSDMD.

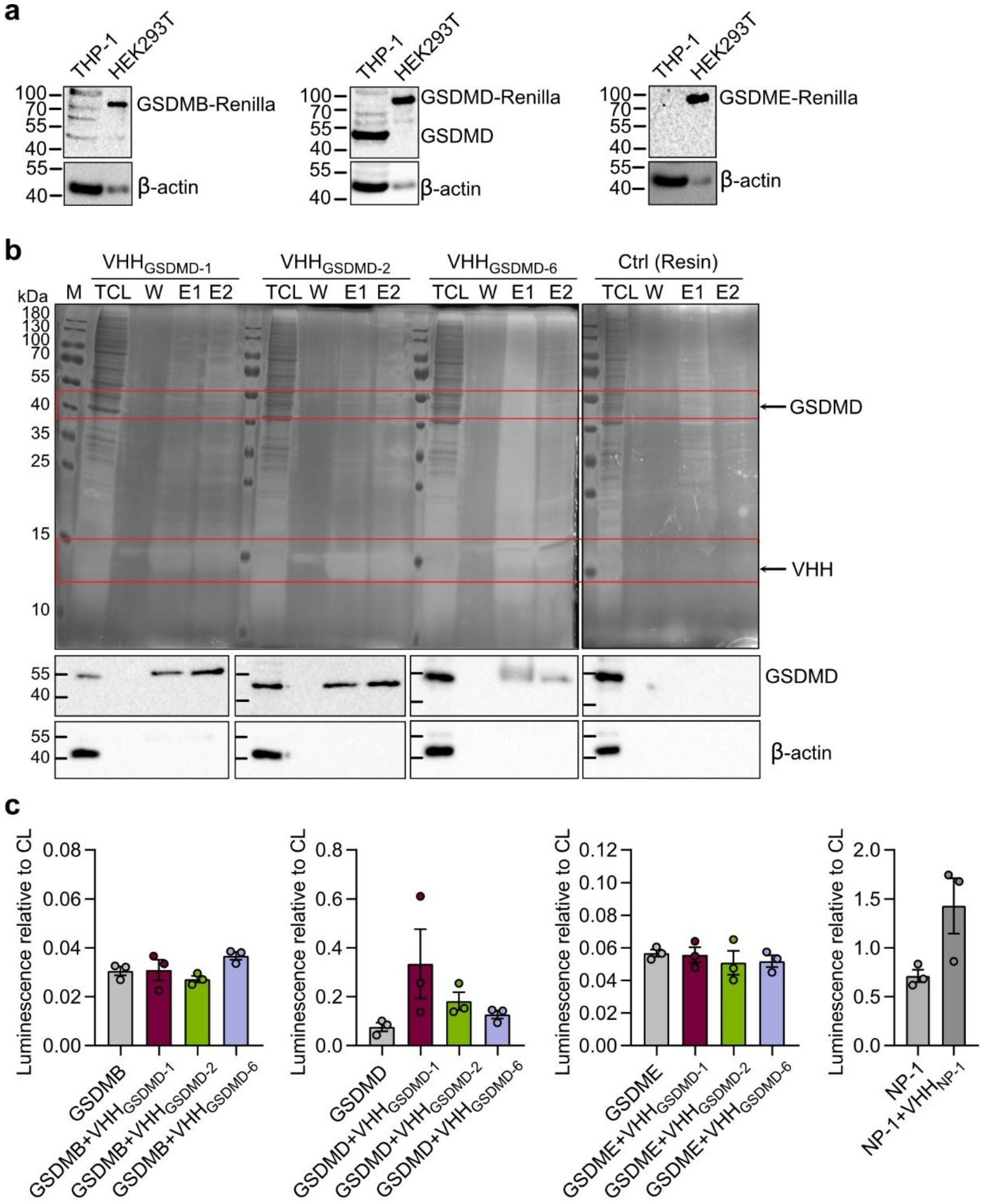

Supplementary Fig. 5 | Specificity of VHH$_{GSDMD-1}$, VHH$_{GSDMD-2}$, and VHH$_{GSDMD-6}$ to human GSDMD. **a**, Western blot analyses of GSDMB, GSDMD and GSDME expression in THP-1 cells and HEK293T transfected with plasmids encoding the gasdermins as renilla-fusion proteins. **b**, A pull-down using CNBr beads coupled to VHH$_{GSDMD-1}$, VHH$_{GSDMD-2}$ and VHH$_{GSDMD-6}$ and THP-1 cell lysate was performed. The protein content of the samples at the different stages of the pull-down was analyzed using SDS-PAGE followed by zinc staining. The samples were further analyzed by western blot using an antibody directed against GSDMD. M: marker, TCL: total cell lysate, W: wash fraction, E1/E2: elution fraction 1/2. **c**, HEK293T cells were transfected with plasmids encoding GSDMB, GSDMD or GSDME as renilla-fusion proteins. Lysates of the transfected cells were combined with recombinant VHH$_{GSDMD-1}$, VHH$_{GSDMD-2}$ or VHH$_{GSDMD-6}$ haboring C-terminal His-tags. A pull-down using an anti-His antibody was performed and renilla luciferase activity was measured as readout for the efficiency of the pull-down. As positive controls a plasmid encoding nucleoprotein of influenza A virus (NP-1) and a corresponding nanobody (VHH$_{NP-1}$) were used. CL: cell lysate. Source data are provided as a Source Data file.

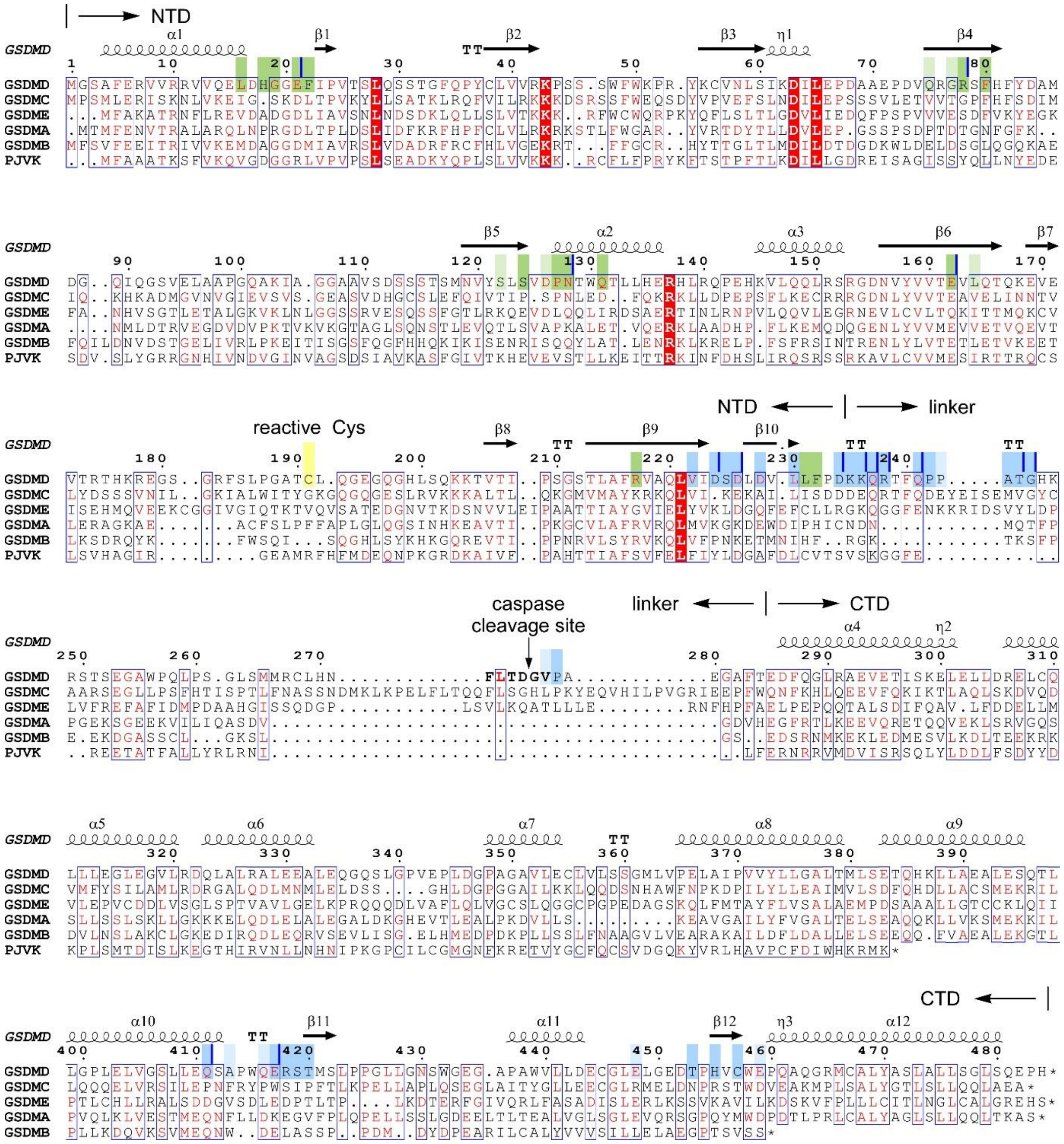

Supplementary Fig. 6 | Sequence alignment of human gasdermin proteins correlated to the secondary structure of GSDMD. Sequence alignment of human gasdermins GSDMA (UniProt ID Q96QA5), GSDMB (Q8TAX9), GSDMC (Q9BYG8), GSDMD (P57764), GSDME (O60443), and PJVK (Q0ZLH3) correlated to the crystal structure of the GSDMD–VHH$_{GSDMD-2}$–VHH$_{GSDMD-6}$ complex determined here (PDB 7z1x). Secondary structure elements of GSDMD, the caspase cleavage site, the reactive Cys191 targetable by covalent inhibitors, and the N- and C-terminal domains are indicated at the top. Residues in red boxes are conserved in all gasdermins, similar residues are marked with red characters. The sequence alignment was performed with MultiAlin and the correlation to secondary structure elements and sequence conservation was done with ESPript 3.0 (ref. 60). Residues in GSDMD mediating direct interactions with VHH$_{GSDMD-2}$ are boxed light and medium green according to the buried surface area (5–12 and >12 Å^2, respectively) as determined with PDBePISA (ref. 59). Similarly, residues in GSDMD mediating direct interactions with VHH$_{GSDMD-6}$ are boxed light and medium blue. Hydrogen-bonds or salt bridges are indicated by a blue bar at the right side of the residue. The low degree of sequence conservation within the human gasdermin family is in agreement with the high specificity of the nanobodies, targeting only human GSDMD.

Supplementary Table 1 | Kinetic rates and dissociation constants of the SPR measurements.

**Binding to human GSDMD** [a]	k_a (1/Ms)	k_d (1/s)	K_D (M)
$VHH_{GSDMD-1}$	$6.48x10^{6}$	$1.98x10^{-3}$	$3.05x10^{-10}$
$VHH_{GSDMD-2}$	$1.49x10^{6}$	$9.51x10^{-4}$	$6.37x10^{-10}$
$VHH_{GSDMD-3}$	$2.80x10^{6}$	$1.54x10^{-3}$	$5.51x10^{-10}$
$VHH_{GSDMD-4}$	$2.89x10^{3}$	$1.61x10^{-3}$	$5.59x10^{-7}$
$VHH_{GSDMD-5}$	$3.02x10^{6}$	$1.49x10^{-3}$	$4.94x10^{-10}$
$VHH_{GSDMD-6}$	$1.41x10^{5}$	$2.01x10^{-2}$	$1.43x10^{-7}$

[a] Association and dissociation rate constants were determined at a flow rate of 30 µl/min. The association step was carried out for 120 s and the dissociation step for 300 s.

Supplementary Table 2 | Crystallographic data collection and refinement statistics.

	GSDMD–$VHH_{GSDMD-2}$–$VHH_{GSDMD-6}$ complex
Data collection [a]	
Beam line	DESY PETRA III P13
Wavelength (Å)	0.976255
Space group	P 3_1
Unit cell: a, b, c (Å)<br>α, β, γ (°)	108.35, 108.35, 124.04<br>90, 90, 120
Resolution range (Å)	93.84–1.86 (1.926–1.86)
Unique reflections	136,585 (13,351)
Multiplicity	2.0 (2.0)
Completeness (%)	99.66 (96.73)
Mean I/sigma(I)	17.43 (0.73)
R_{meas}	0.023 (1.209)
$CC_{1/2}$	1.0 (0.443)
Reflections used in refinement	136,351 (13,167)
Reflections used for R-free	1997 (193)
Refinement	
Model content	A, D: GSDMD (1-484, Δ184-194, Δ247-272)<br>B, E: $VHH_{GSDMD-2}$ (1-118)<br>C, F: $VHH_{GSDMD-6}$ (1-123)
# of atoms macromolecules	9700
# of ligands	0
# of solvent	557
R_{work}	0.2124 (0.3297)
R_{free}	0.2493 (0.3204)
RMS deviations bonds [Å]	0.008
RMS deviations angles [°]	0.79
Ramachandran favored (%)	96.24
Ramachandran allowed (%)	3.35
Average B-factor	49.55
Macromolecules	49.621
ligands	–
solvent	48.37
PDB accession code	7z1x

[a] Values in parentheses are for the highest resolution shell.
R_{free}-value is equivalent to the R-value but is calculated for 5% of the reflections chosen at random and omitted from the refinement process.
