# Supplementary material for: Pyroptosis inhibiting nanobodies block Gasdermin D pore formation
Source: Nat Commun. 2023 Dec 1;14:7923. doi: 10.1038/s41467-023-43707-z (PMC10692205; doi:10.1038/s41467-023-43707-z)
Supplement: Supplementary file 3 — Reporting Summary [file 41467_2023_43707_MOESM3_ESM.pdf]

nature portfolio

Corresponding author(s): Matthias Geyer

Last updated by author(s): Oct 12, 2023

Reporting Summary

Nature Portfolio wishes to improve the reproducibility of the work that we publish. This form provides structure for consistency and transparency in reporting. For further information on Nature Portfolio policies, see our Editorial Policies and the Editorial Policy Checklist.

Statistics

For all statistical analyses, confirm that the following items are present in the figure legend, table legend, main text, or Methods section.

n/a	Confirmed	
☐	☒	The exact sample size (*n*) for each experimental group/condition, given as a discrete number and unit of measurement
☐	☒	A statement on whether measurements were taken from distinct samples or whether the same sample was measured repeatedly
☒	☐	The statistical test(s) used AND whether they are one- or two-sided<br>*Only common tests should be described solely by name; describe more complex techniques in the Methods section.*
☒	☐	A description of all covariates tested
☒	☐	A description of any assumptions or corrections, such as tests of normality and adjustment for multiple comparisons
☐	☒	A full description of the statistical parameters including central tendency (e.g. means) or other basic estimates (e.g. regression coefficient) AND variation (e.g. standard deviation) or associated estimates of uncertainty (e.g. confidence intervals)
☒	☐	For null hypothesis testing, the test statistic (e.g. *F*, *t*, *r*) with confidence intervals, effect sizes, degrees of freedom and *P* value noted<br>*Give P values as exact values whenever suitable.*
☒	☐	For Bayesian analysis, information on the choice of priors and Markov chain Monte Carlo settings
☒	☐	For hierarchical and complex designs, identification of the appropriate level for tests and full reporting of outcomes
☒	☐	Estimates of effect sizes (e.g. Cohen's *d*, Pearson's *r*), indicating how they were calculated

Our web collection on statistics for biologists contains articles on many of the points above.

Software and code

Policy information about availability of computer code

Data collection	X-ray crystallography data were collected at beamline P13 of the PETRA III synchrotron at the “Deutsches Elektronen-Synchrotron” (DESY) (Hamburg, Germany) at a wavelength of 0.976255 A.
Data analysis	X-ray crystallography data were analysed using PyMOL 2.0, Coot 0.9, PHENIX 1.19.1, XDS Version January 31, 2020 and MolProbity 4.5.1. SPR data were analysed with the Biacore Insight Evaluation Software (version 3.0.12.15655). Multi-angle light scattering (MALS) data analysis was done using the ASTRA V software (WYATT). Nano differential scanning fluorimetry (nanoDSF) data were analysed using the software PR.ThermControl version 2.1.5 (NanoTemper). SDS-gels and blots were analysed using ChemiDoc XRS+ system ImageLab (v.6.0.1) (BioRad). Grpahs were plotted using GraphPad Prism (v.7).

For manuscripts utilizing custom algorithms or software that are central to the research but not yet described in published literature, software must be made available to editors and reviewers. We strongly encourage code deposition in a community repository (e.g. GitHub). See the Nature Portfolio guidelines for submitting code & software for further information.

Data

Policy information about availability of data

All manuscripts must include a data availability statement. This statement should provide the following information, where applicable:

- Accession codes, unique identifiers, or web links for publicly available datasets
- A description of any restrictions on data availability
- For clinical datasets or third party data, please ensure that the statement adheres to our policy

The authors declare that the data supporting the findings of this study are available within the paper and its supplementary information files. Source data are provided with this paper. Structure coordinates and diffraction data of the human GSDMD–VHHGSDMD-2–VHHGSDMD-6 complex were deposited in the Protein Data Bank (http://www.pdb.org) under accession codes 7z1x [http://doi.org/10.2210/pdb7Z1X/pdb]. The coordinate data used in this study are available in the PDB database under accession codes 5ivo [http://doi.org/10.2210/pdb5IVO/pdb], 6n9o [http://doi.org/10.2210/pdb6n9o/pdb], 6vfe [http://doi.org/10.2210/pdb6vfe/pdb].

Research involving human participants, their data, or biological material

Policy information about studies with human participants or human data. See also policy information about sex, gender (identity/presentation), and sexual orientation and race, ethnicity and racism.

Reporting on sex and gender	n/a
Reporting on race, ethnicity, or other socially relevant groupings	n/a
Population characteristics	n/a
Recruitment	n/a
Ethics oversight	n/a

Note that full information on the approval of the study protocol must also be provided in the manuscript.

Field-specific reporting

Please select the one below that is the best fit for your research. If you are not sure, read the appropriate sections before making your selection.

☒ Life sciences ☐ Behavioural & social sciences ☐ Ecological, evolutionary & environmental sciences

For a reference copy of the document with all sections, see nature.com/documents/nr-reporting-summary-flat.pdf

Life sciences study design

All studies must disclose on these points even when the disclosure is negative.

Sample size	No sample size calculations were performed. No experients involving biological specimens were examined such that sample size does not apply. 18 crystals were screend to identify the optimal crystal for data collection. The number of crystals screened was random and was not limited by any experimental parameter. For surface plasmon resonance (SPR) experiments, optimal analyte concentrations were determined in initial screens, before binding affinities were determined in a single experiment.<br>Biochemical experiments were confirmed with multiple biological replicates as detailed in the Methods and Figure Legends.
Data exclusions	No data were excluded from analyses.
Replication	Crystals could be reproducibly grown and structures were determined at different resolutions. All other experiments were confirmed with multiple biological replicates as detailed in the Methods and Figure legends.
Randomization	No experiment involving animals or humans was performed in this study, therefore randomization is not applicable for this study.
Blinding	Blinding is not applicable for this study as no experiment involving humans or animals was performed.

Reporting for specific materials, systems and methods

We require information from authors about some types of materials, experimental systems and methods used in many studies. Here, indicate whether each material, system or method listed is relevant to your study. If you are not sure if a list item applies to your research, read the appropriate section before selecting a response.

Materials & experimental systems

n/a	Involved in the study
[ ]	[x] Antibodies
[ ]	[x] Eukaryotic cell lines
[x]	[ ] Palaeontology and archaeology
[x]	[ ] Animals and other organisms
[x]	[ ] Clinical data
[x]	[ ] Dual use research of concern
[x]	[ ] Plants

Methods

n/a	Involved in the study
[x]	[ ] ChIP-seq
[x]	[ ] Flow cytometry
[x]	[ ] MRI-based neuroimaging

Antibodies

Antibodies used	Primary antibodies: rabbit anti-GSDMB (Cell Signaling Cat#76349, RRID:AB_ 2799883), r rabbit polyclonal anti-GSDMD (Atlas Antibodies Cat# HPA044487, RRID:AB_2678957), rabbit anti-DFNA5/GSDME clone EPR19859 (Abcam Cat# ab215191, RRID:AB_2737000), mouse anti-6x-His-Tag (Thermo Fisher Scientific Cat#MA1-135, RRID:AB_2536841), mouse anti-β-actin (Santa Cruz Biotechnology Cat #sc-47778, RRID:AB_626632)<br>Secondary antibodies: mouse anti-m-IgGk BP-HRP (Santa Cruz Biotechnology Cat #sc-516102, RRID:AB_2687626,), mouse anti-rabbit IgG-HRP (Santa Cruz Biotechnology Cat #sc-2357, RRID:AB_628497)
Validation	All commercial antibodies were verified according to manufacturer's specifications on their corresponding websites.

Eukaryotic cell lines

Policy information about cell lines and Sex and Gender in Research

Cell line source(s)	Human embryonic kidney (HEK) 293T cells (ATCC Cat# CRL-3216, RRID: CVCL_0063), THP-1 cells (ATCC TIB-202)
Authentication	Cell lines were verfieid by manufacturer's website and cellular identity was regularly checked by morphology.
Mycoplasma contamination	Cell lines were tested mycoplasma negative through PCR.
Commonly misidentified lines<br>(See ICLAC register)	No commonly misidentified cell lines were used.
