# Supplementary material for: Pyroptosis inhibiting nanobodies block Gasdermin D pore formation
Source: Nat Commun. 2023 Dec 1;14:7923. doi: 10.1038/s41467-023-43707-z (PMC10692205; doi:10.1038/s41467-023-43707-z)

Source Data Fig. 5, Supplementary Fig. 1 | Uncropped Blots

Fig. 5a

GSDMD+Caspase-4

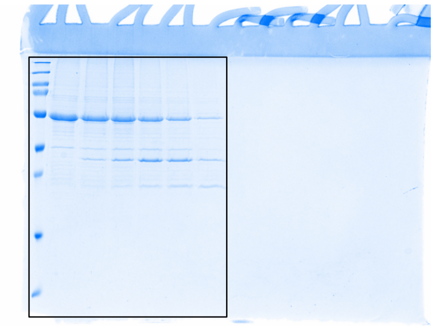

GSDMD+Caspase-4+VHH$_{GSDMD-1}$

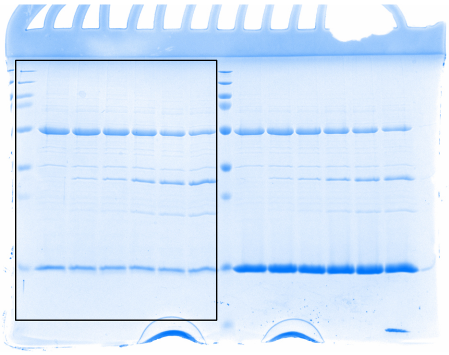

GSDMD/VHH ratio 1:1

GSDMD+Caspase-4+VHH$_{GSDMD-2}$

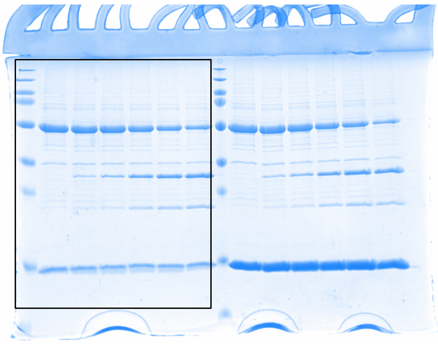

GSDMD/VHH ratio 1:1

Supplementary Fig. 1b

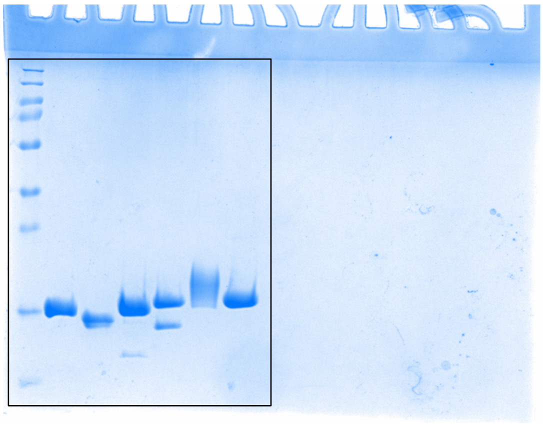

Supplementary Fig. 1c

human GSDMD wt, f.l.

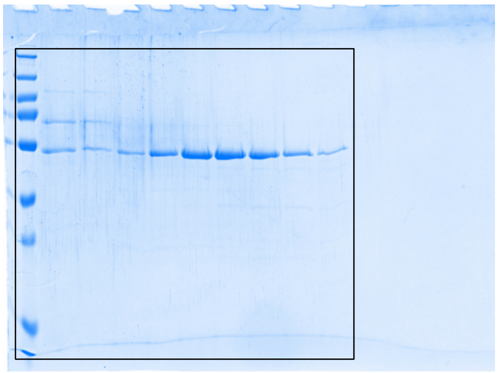

Supplementary Fig. 1d

hGSDMD (Δ184-194/Δ247-272)

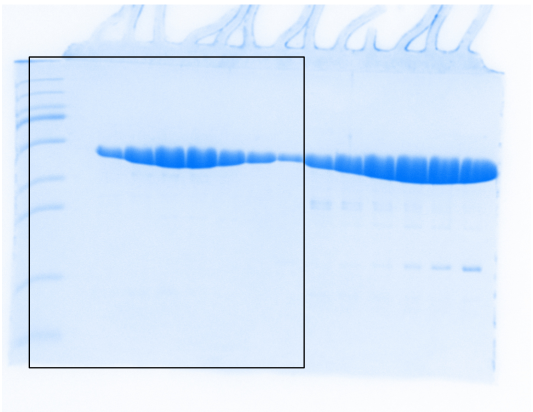

Supplementary Fig. 1e

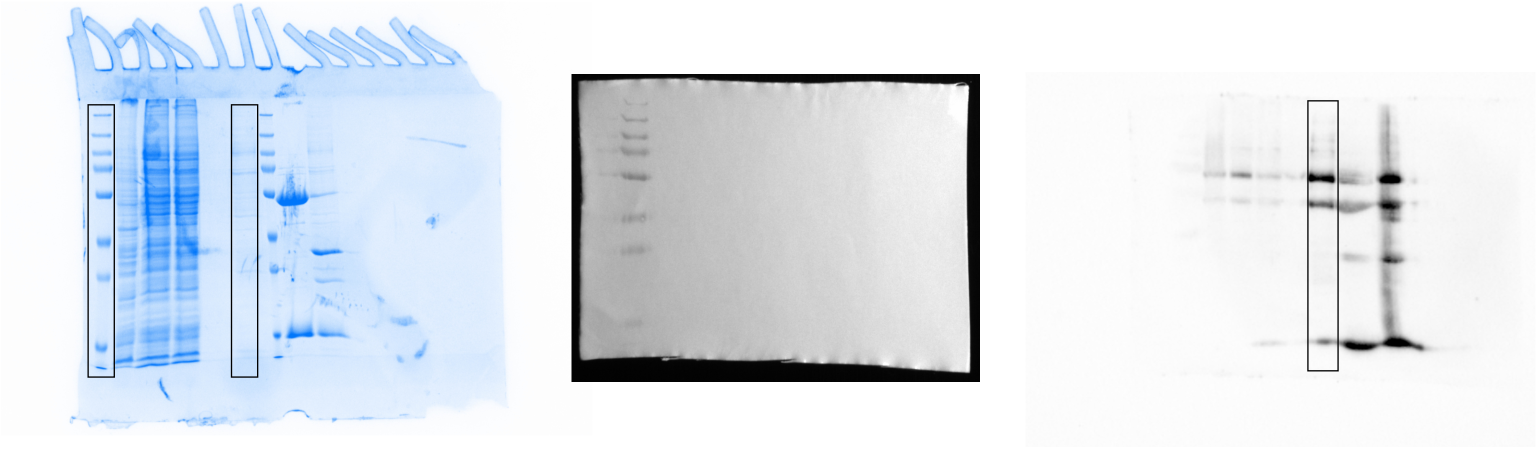

Supplement: Supplementary file 4 — Source Data [file 41467_2023_43707_MOESM4_ESM.zip › Kopp et al Source Data/Source Data Fig.5a Supplementary Fig.1bcde.pdf]
