# Supplementary figures and images for: Pyroptosis inhibiting nanobodies block Gasdermin D pore formation
Source: Nat Commun. 2023 Dec 1;14:7923. doi: 10.1038/s41467-023-43707-z (PMC10692205; doi:10.1038/s41467-023-43707-z)

Source Data Supplementary Fig. 5a | Uncropped Blots

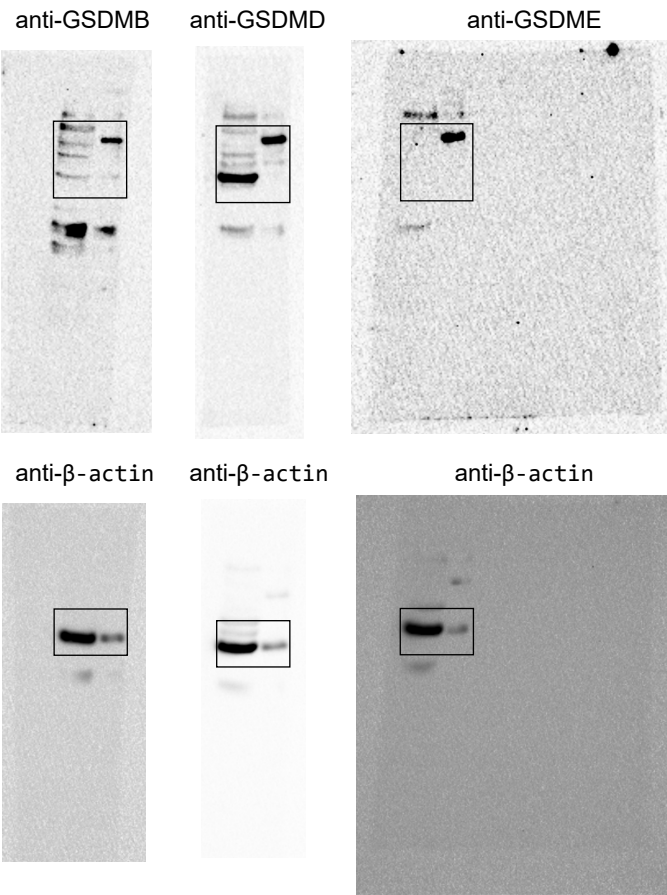

Supplement: Supplementary file 4 — Source Data [file 41467_2023_43707_MOESM4_ESM.zip › Kopp et al Source Data/Source Data Supplementary Fig.5a.pdf]

Source Data Supplementary Fig. 5b | Uncropped Blots

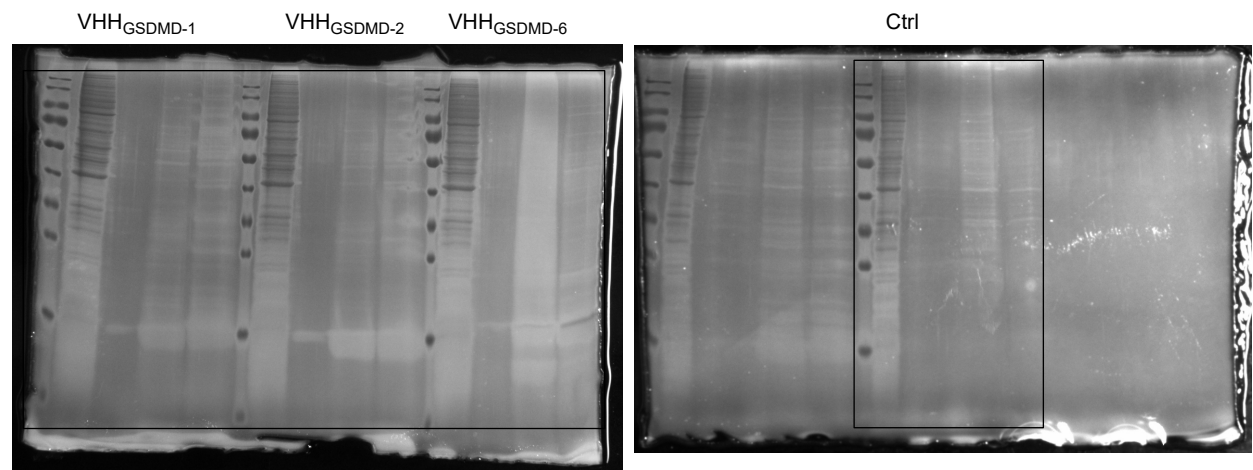

anti-GSDMD

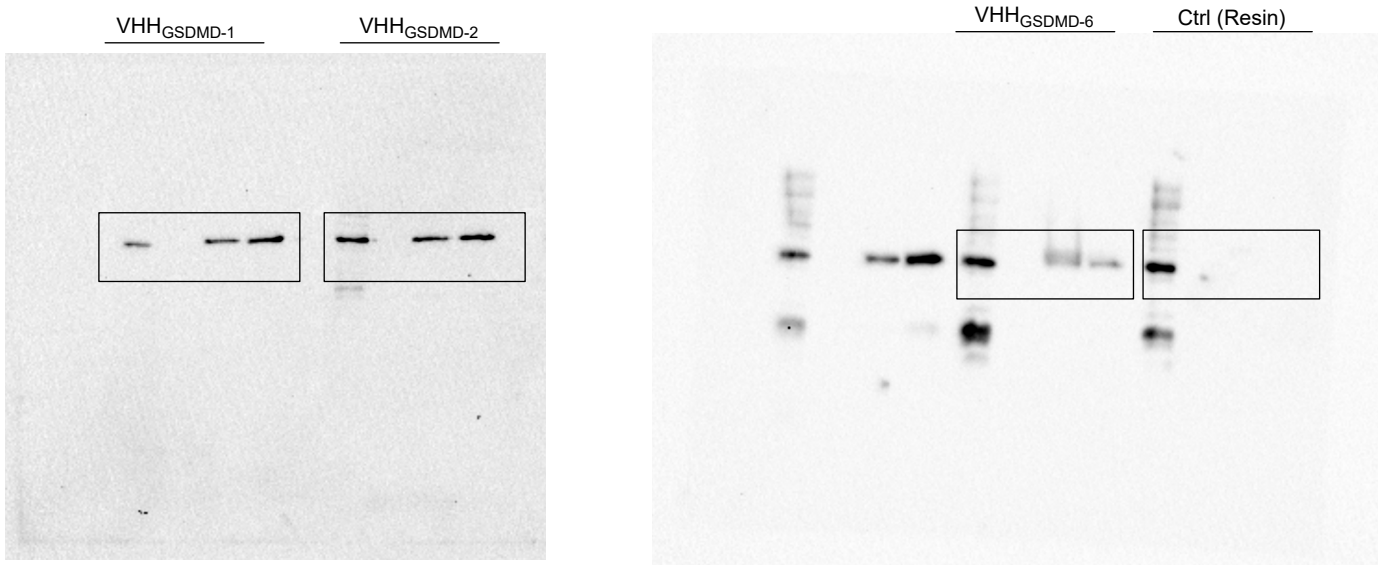

anti-β-actin

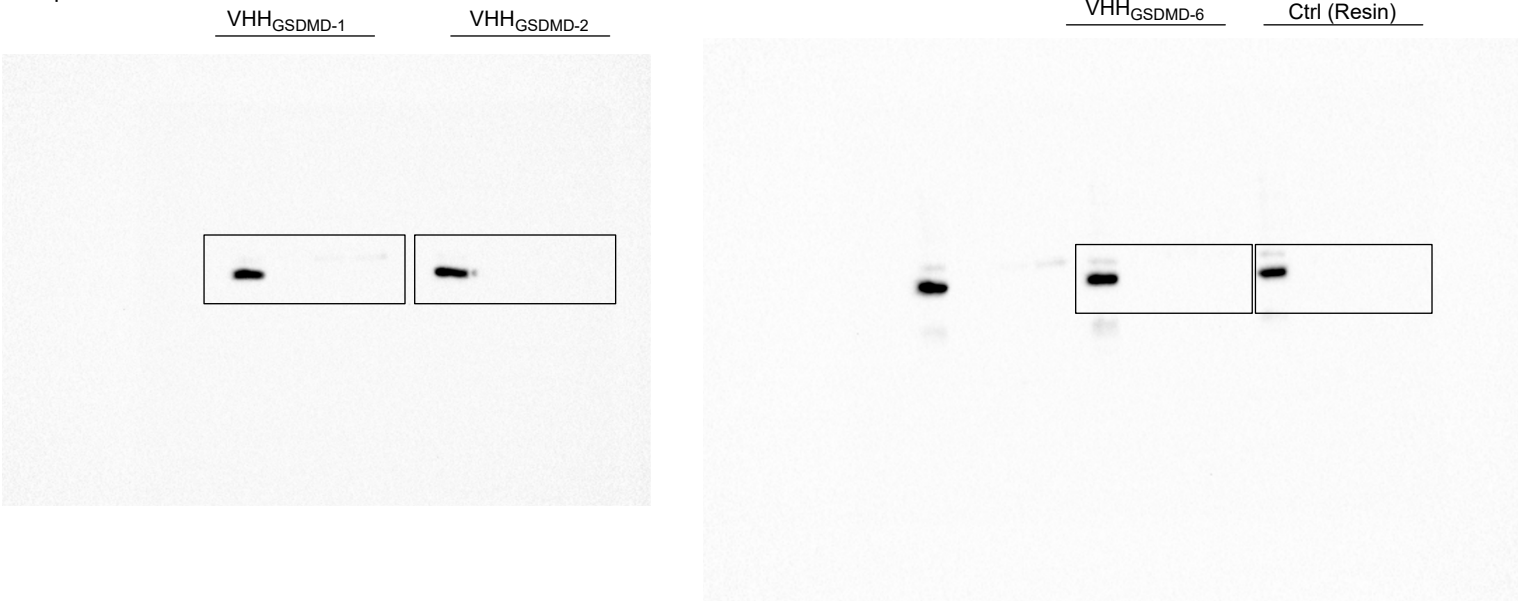

Supplement: Supplementary file 4 — Source Data [file 41467_2023_43707_MOESM4_ESM.zip › Kopp et al Source Data/Source Data Supplementary Fig.5b.pdf]
